# REGAIN STUDY: Retrospective Study to Assess the Effectiveness, Tolerability, and Safety of Ferric Carboxymaltose in the Management of Iron Deficiency Anemia in Pregnant Women

**DOI:** 10.1155/2019/4640635

**Published:** 2019-11-12

**Authors:** Saleema Wani, Mariyam Noushad, Shabana Ashiq

**Affiliations:** Obstetrics & Gynaecology Department, Corniche Hospital, P.O. Box 3788, Abu Dhabi, UAE

## Abstract

Iron deficiency anemia (IDA) during pregnancy arises because of preexisting inadequate stores or complex physiological changes and can lead to serious maternal and fetal complications. Oral iron, either as iron sulfate or fumarate, with or without folic acid, is the most commonly used treatment for IDA in pregnancy. Intravenous (IV) iron has a role in the treatment of IDA in pregnancy, particularly in women who present late, display severe anemia (Hb ≤ 9 g/dL), or risk factors, and are intolerant/noncompliant of oral iron. Previously, administration of IV iron was minimal, owing to potentially serious anaphylactic reactions. Recently, new IV iron products have been developed, offering better compliance, tolerability, efficacy, and a good safety profile. Our study aimed to assess the effectiveness, safety, and tolerability of IV ferric carboxymaltose (FCM) in the treatment of IDA in pregnant women in the UAE. Data from 1001 pregnant women who received at least one administration of FCM (500, 1000, or 1500 mg) during their second or third trimester of pregnancy (2 years backward from study initiation) were collected retrospectively from electronic medical records at Corniche Hospital, Abu Dhabi, UAE. Results showed that 41.4% of the women were able to achieve an increase of ≥2 g/dL in blood hemoglobin overall. A change of ≥2 g/dL was achieved by 27.5% of women administered a dose of 500 mg, 39.2% of women administered a dose of 1000 mg, and 63.9% of women administered a dose of 1500 mg of IV FCM. This indicates a directly proportional relationship between increasing IV FCM dose and the increase of ≥2 g/dL in blood hemoglobin. A total of 7 (0.7%) women reported mild, nonserious adverse events during the study. Within the limits of this retrospective study, IV FCM therapy was safe and effective in increasing the mean hemoglobin of pregnant women with IDA.

## 1. Introduction

The World Health Organization (WHO) defines iron deficiency anemia (IDA) as hemoglobin (Hb) < 12.0 g/dL in women aged >15 years and <11.0 g/dL in pregnant women [1]. The presence of IDA during pregnancy depends on two factors: the woman's preexisting iron stores during conception and the amount of iron absorbed during gestation. IDA during pregnancy increases perinatal risks for mothers and neonates, and increases the overall infant mortality. One of the classic laboratory findings of IDA includes a decrease in the Hb level: <11 g/dL during the 1^st^ trimester, <10.5 g/dL during the 2^nd^ trimester, and <11 g/dL during the 3^rd^ trimester are diagnostic for anemia during pregnancy [2].

Prophylactic oral iron therapy is given to all pregnant women with normal hemoglobin values throughout pregnancy. However, IV iron therapy is recommended during the 2^nd^ and 3^rd^ trimesters for women with severe anemia (Hb < 9 g/dL) and risk factors (coagulation disorders and placenta previa), emergency situations that require prompt resolution of anemia (paleness, tachycardia, tachypnea, syncope, heart failure, respiratory failure, angina pectoris, and signs of cerebral hypoxia), and noncompliance to oral iron because of intolerance (gastrointestinal adverse events) [3, 4]. The threshold of Hb that warrants treatment with IV iron differs by country and is higher in Asia-Pacific countries.

High molecular weight iron dextran, the first IV iron product from the mid-20^th^ century, was associated with high and eminent risk of anaphylactic reactions [5], dissuading physicians from using IV iron for the treatment of IDA for many years. Iron sucrose and ferric carboxymaltose (FCM) are dextran-free IV iron alternatives. FCM permits single dose, short 15-minute infusions, and higher dosing (up to 1000 mg), making it an attractive alternative in terms of safety, efficacy, convenience, and resource utilization [6–9]. The most commonly reported adverse drug reactions (ADR) during clinical studies (>8,000 subjects who received FCM) and from postmarketing experience were nausea (occurring in 2.9% of the subjects), followed by injection/infusion site reactions, hypophosphatemia, headache, flushing, dizziness, and hypertension. The most serious ADR is anaphylactoid/anaphylactic reactions (rare), and the risk is enhanced for patients with known allergies (drug allergies, history of severe asthma, eczema, or other atopic allergies), and patients with immune or inflammatory conditions (systemic lupus erythematosus and rheumatoid arthritis) [10].

In a prospective observational study in 65 anemic pregnant women, FCM (up to 15 mg/kg) given between 24 and 40 weeks of pregnancy significantly increased Hb (*p* < 0.01) above baseline levels in all women with no serious side effects [11].

A systematic review of 21 randomized controlled trials and 26 observational studies to demonstrate the safety and efficacy of 3 commonly used IV iron preparations (iron polymaltose (IPM), ferric carboxymaltose (FCM), or iron sucrose (IS)) in pregnancy showed that the median prevalence of adverse drug reactions from IPM, FCM, and IS was 2.2% (range: 0–4.5%), 5.0% (range: 0–20%), and 6.7% (range: 0–19.5%), respectively [12].

Rather, a few cases with limited numbers of FCM-treated pregnant women have been reported in the UAE. Thus, our study aimed to assess the effectiveness, safety, and tolerability of IV FCM in the treatment of IDA in pregnant women in the UAE.

## 2. Materials and Methods

This was a retrospective study that included data of 1001 pregnant women (2 years backward from the date of study initiation) from the electronic medical records of Corniche Hospital, Abu Dhabi in the UAE. Data of women aged 18 to 45 years, with a confirmed pregnancy (pregnancy test, *β*-human chorionic gonadotropin), who received at least one administration of FCM (Ferinject®) during their second or third trimester of pregnancy for the treatment of IDA, with at least two Hb readings (before and after FCM administration), and with a Hb value <11 g/dL, were included in this study.

Data of pregnant women with hematological or hemopoietin disorders other than IDA, and/or those receiving parenteral iron other than FCM, earlier in pregnancy, were excluded from the study. The data extracted were examined to identify subgroups of women who received different doses of FCM infusion (500 mg, 1000 mg, and 1500 mg), and changes in hemoglobin levels in the blood were compared across these subgroups.

### 2.1. Statistical Analysis

With the assumption that 30% of the women would drop out during the study, the number of required women was 980 rounded to 1000 women (confidence level 95%; alpha error level = 0.05). The analysis of the primary efficacy endpoints mean change in blood hemoglobin post-FCM infusion was performed by the paired *t*-test, and the percentage of women achieving a change of 2 g/dL in blood Hb level from baseline was presented with a 95% confidence interval. The expected increase in the hemoglobin level was anticipated to be 1.34 g/dL. With type I error of 5% (two-sided) and a power of 90%, 784 women were needed. The AEs were grouped by body system and presented with descriptive statistics, numbers, and percentages with their 95% confidence intervals.

## 3. Results and Discussion

The data of 1001 pregnant women with IDA from the electronic medical records of Corniche Hospital in the UAE were eligible for the analysis of this study. A total of 93.9% (*n* = 940) of the women had a single foetus, 5.6% (*n* = 56) had a twin pregnancy, and 0.5% (*n* = 5) had triplets or quadruplets.

### 3.1. Demographics and Baseline Characteristics

The mean ± SD age of the women (*N* = 1001) included in this analysis was 31.83 ± 5.80 (range: 18.85–45.70) years. The majority of the women were Arab (94.6%), and the rest were Asian (3.6%), African (0.9%), and others (0.4%). The mean ± SD weight of the women in this analysis was 75.95 kg ± 15.49, their mean ± SD systolic and diastolic blood pressure was 109.71 ± 10.77 and 66.85 ± 8.23 mmHg, respectively. Their mean ± SD heart rate was 90.49 ± 11.23 beats per minute (Table 1).

A total of 355/793 women had medical conditions, the most frequent being gestational diabetes (32.11%), followed by hypothyroidism (21.97%), asthma (12.68%), type 2 diabetes mellitus (7.04%), IDA (3.38%), and migraine (3.10%).

A total of 55.1% (441/801) women underwent surgery, the most common being cesarean section (71.20%), followed by gastrectomy (9.30%) and uterine dilation/curettage (5.22%).

A total of 32% (237/741) of the women had taken at least one concomitant medication preinfusion, and 29.7% (187/628) took concomitant medication at the postinfusion time point (listed in supplementary materials). The mean ± SD Hb and serum ferritin level were 9.38 ± 0.82 g/L and 7.92 ± 3.54 pmol/L, respectively.

Majority (70.3%) of the women received 1000 mg FCM infusion, 15.5% received 1500 mg FCM infusion, and 14.2% received 500 mg FCM infusion. Blood Hb levels were assessed at the postinfusion time point (Table 2).

#### 3.1.1. Efficacy Analyses

Results showed that the mean levels of Hb significantly increased (*p* < 0.0001) from 9.38 g/L to 11.16 g/L; this is an increase of 1.772 g/L (95% CI: 1.705–1.839). Furthermore, the proportion of women achieving a change of at least 2 g/dl in blood Hb level from the baseline was found to represent 41.4% (*n* = 414) of the population.

#### 3.1.2. Subset Analyses

Changes in the mean blood Hb levels from pre to postinfusion were further analyzed by the different doses of FCM infused (500, 1000, and 1500 mg); this indicates a significant increase in hemoglobin levels in the blood (Table 3).

Moreover, the higher the dose of FCM, the greater the proportion of women who achieved a change ≥2 g/dl in blood Hb (Table 4). About 27.5% of women taking a dose of 500 mg achieved a change of ≥2 g/dl, 39.2% of women taking a dose of 1000 mg achieved a change of ≥2 g/dl, and 63.9% of women taking a dose of 1500 mg achieved a change of ≥2 g/dl.

#### 3.1.3. Safety Analyses

A total of 7 (0.7%) women experienced AEs mainly, rash, pruritus, and allergic dermatitis. Five of these AEs were mild and two were missing information, but none were serious. A total of 6/7 women suspected that the AE was due to the FCM; of these, 4 received therapy for the AE, and all six eventually recovered. Only one woman permanently discontinued FCM (Table 5).

#### 3.1.4. Discussion

This retrospective study investigated the efficacy and safety of IV FCM to treat IDA during pregnancy. FCM treatment substantially increased Hb in 50% of women with IDA from baseline to postinfusion. There were no anaphylactic reactions or any serious adverse events reported with FCM treatment across all three dose groups.

Our results concur with other studies which have shown the safe and efficient use of FCM in pregnancy and postpartum. A retrospective case-controlled study to assess the safety and efficacy of IV FCM in 128 pregnant women with IDA versus the control group (nonanemic or low-grade anemia) showed that IV FCM was a safe and effective treatment [13].

The results from our study are in agreement with the outcomes from several randomized controlled trials of IV FCM. In a study that assessed efficacy and safety of IV FCM versus oral ferrous sulfate (FS), FCM achieved Hb increase of >2 g/dL in 7 days and >3 g/dL in 2 to 4 weeks [14]. Another study comparing the efficacy and safety of IV FCM versus oral FS in pregnant Korean women (gestational weeks: 16–33) with IDA showed that FCM provided significantly greater improvements in iron parameters and quality of life (QoL) than FS [15].

A study in 246 pregnant women (mean gestation: 27 weeks) that compared the efficacy and safety of 1000 mg IV FCM (*n* = 83) against 325 mg oral FS (*n* = 81) and 1000 mg IV iron polymaltose (IPM) (*n* = 82) for the treatment of IDA in pregnancy showed similar results; administration of IV FCM during pregnancy was safe and better tolerated than oral FS or IV IPM and a significant improvement in the overall QoL scores was observed in both IV iron supplement groups by achieving normal ferritin following effective and prompt repletion of iron stores, compared with the oral iron group (*p*=0.04, 95% CI: 21.3, 1.8). There were no differences in the fetal outcomes between the three trial arms. FCM was more convenient than other treatments [16].

A retrospective study which investigated the efficacy and safety of IV FCM versus IV iron dextran in 92 pregnant women with IDA supported the results of our study, showing that IV FCM was effective and safe, with low risk of serious adverse events [17]. Another retrospective analysis which compared the efficacy of IV FCM versus IV iron sucrose in 206 pregnant women with IDA and intolerant to oral iron showed that FCM was the treatment of choice, owing to its safety and efficacy [18].

Also, a randomized study compared IV FCM and iron sucrose (IS) for 12 weeks in 200 pregnant women with IDA showed that IV FCM improved laboratory biomarkers (Hb, mean corpuscular volume, serum iron, serum ferritin, total iron-binding capacity, and transferrin saturation), symptoms, and health-related QoL score in a shorter duration of time as compared with IS [19]. In another randomized trial, pregnant women diagnosed with moderate to severe IDA received either 1000 mg IV FCM (*n* = 50) or 600 mg intravenous iron sucrose complex (ISC) (*n* = 50). Mean rise in Hb at 12 weeks was significantly higher in FCM group (29 g/L vs 22 g/L; *p* value < 0.01). FCM was associated with greater improvement in fatigue scores. No serious adverse events were noted in either group. Treatment with FCM resulted in rapid replenishment of iron stores in pregnant women with significantly higher Hb rise over the period of 12 weeks [20]. Similarly, a study of 200 women of postpartum iron deficiency anemia who were randomized into two groups, I.V. iron sucrose in multiple doses, 200 mg/day, total of 1000 mg and I.V. ferric carboxymaltose 1000 mg single dose, showed that there was a statistically significant increase (*p* < 0.001) in Hb in the FCM group 4.68 g/dL compared with the iron sucrose group 3.92 g/dL [21].

In addition, an observational prospective study in pregnant women (12–34 weeks gestation, Hb 7 to 9.9 g/dL) compared FCM infusion (*n* = 30) with iron sucrose infusion (IS) (*n* = 30), mean increase in Hb levels posttreatment in group IS was 2.35 ± 0.41 vs. 2.52 ± 0.073 in group FCM (*p* < 0.0000001). Side effects were noted in 36.7% of the women in group IS vs 33.3% of the women in group FCM (*p* > 0.05). Intravenous FCM administration in pregnancy is likely to be safe and effective [22].

Thus, intravenous FCM is a safe agent in pregnancy and is noninferior to oral iron or other parenteral iron preparations for the treatment of IDA in pregnancy.

## 4. Conclusions

Nearly 50% of the women in this study achieved an increase of ≥2 g/dl in blood hemoglobin without serious adverse effects. Within the limits of this retrospective study, IV FCM therapy was safe and effective in increasing blood hemoglobin levels in pregnant women with IDA in the 2^nd^ and 3^rd^ trimesters.

## Figures and Tables

**Table 1:** Body weight and vital signs (pre- and postinfusion timepoint).

		Weight (kg)	Systolic blood pressure (mmHg)	Diastolic blood pressure (mmHg)	Heart rate (beats/min)
Preinfusion timepoint					
*N*	Valid	995	996	996	991
	Missing	6	5	5	10
Mean		75.95	109.71	66.85	90.49
Std. deviation		15.49	10.77	8.23	11.23
Range (min–max)		115 (38–153)	114 (81–195)	61 (37–98)	77 (56–133)

Postinfusion timepoint					
*N*	Valid	250	904	903	900
	Missing	751	97	98	101
Mean		73.32	105.69	64.58	88.01
Std. deviation		15.46	10.63	8.37	10.44
Range (min–max)		81 (44–126)	90 (84–174)	54 (44–98)	58 (60–118)

**Table 2:** Clinical laboratory parameters.

		Hemoglobin	Serum ferritin
*N*	Valid	1001	998
	Missing	0	3
Mean ± SD		9.38 ± 0.82	7.92 ± 3.54
Range (min–max)		5 (6–11)	50 (3–53)

**Table 3:** Mean change of hemoglobin from pre- to postinfusion by FCM doses.

FCM dose (mg)	Mean	*N*	Std. deviation	Std. error mean	*p* value
500					
Preinfusion	9.91	142	0.53	0.04	
Postinfusion	11.40	142	0.95	0.08	<0.0001

1000					
Preinfusion	9.42	704	0.83	0.03	
Postinfusion	11.12	704	1.04	0.03	<0.0001

1500					
Preinfusion	8.76	155	0.63	0.05	
Postinfusion	11.10	155	0.97	0.07	<0.0001

**Table 4:** Proportion of patients with a change of ≥2 g/dl in blood hemoglobin by FCM doses.

FCM dose (mg)	Frequency				Percent
500		
Less than 2	103				72.5
≥2	39				27.5
Total	142				100.0

1000		
Less than 2	428				60.8
≥2	276				39.2
Total	704				100.0

1500		
Less than 2	56				36.1
≥2	99				63.9
Total	155				100.0

**Table 5:** Adverse events.

	Grade	Serious	Received therapy	Suspected because of drug	Discontinued drug	Recovered
1	Mild	No	No	Suspected	No	Yes
2	Mild	No	Yes	Suspected	Yes	Yes
3	Mild	No	No	Suspected	No	Yes
4	Unknown	No	Yes	Suspected	No	Yes
5	Mild	No	Yes	Suspected	No	Yes
6	Mild	No	Yes	Suspected	No	Yes

## Data Availability

The tables used to support the findings of this study are included within the article and supplementary materials.

## Supplementary Materials

Supplementary Materials Listing of concomitant medications.
