# Supplementary material for: REGAIN STUDY: Retrospective Study to Assess the Effectiveness, Tolerability, and Safety of Ferric Carboxymaltose in the Management of Iron Deficiency Anemia in Pregnant Women
Source: Anemia. 2019 Nov 12;2019:4640635. doi: 10.1155/2019/4640635 (PMC6875230; doi:10.1155/2019/4640635)
Supplement: Supplementary Materials — Listing of concomitant medications. [file 4640635.f1.pdf]

Supplementary Materials

Listing of Concomitant Medications

	Frequency	Percent
Metformin	68	19.54
Levothyroxine	67	19.25
Ferrous Sulfate/Folic acid	33	9.48
Multivitamins	22	6.32
Insulin	15	4.31
Insulin Detemir	11	3.16
Vitamin D	11	3.16
Budesonide/Formoterol	9	2.59
Ferrous sulfate	9	2.59
Insulin Aspart	5	1.44
Labetalol	5	1.44
Salbutamol	5	1.44
Chlorphenamine	4	1.15
Metformin XR	4	1.15
Acetylsalicylic acid	3	0.86
Al/Mg hydroxide	3	0.86
Albuterol	3	0.86
Calcium	3	0.86
Enoxaparin sodium	3	0.86
Ergocalciferol	3	0.86
Hydrocortisone	3	0.86
Prednisolone	3	0.86
Ranitidine	3	0.86
Amitriptyline	2	0.57
Azathioprine	2	0.57
Bisoprolol	2	0.57
Folic acid	2	0.57
Hydroxychloroquine	2	0.57
Levetiracetam	2	0.57
Methyldopa	2	0.57
Sertraline	2	0.57
Amoxicillin/Clavulanic acid	1	0.29
Ampicillin	1	0.29
Azithromycin	1	0.29
Betamethasone	1	0.29
Budesonide	1	0.29
Cabergoline	1	0.29
Calcium/Vitamin D	1	0.29
Carbamazepine	1	0.29

7	-Frequency	-Percent
Carbimazole	1	0.29
Cefixime	1	0.29
Ceftriaxone	1	0.29
Clonazepam	1	0.29
Clotrimazole	1	0.29
Cyanocobalamin	1	0.29
Cyclobenzaprine	1	0.29
Ferrous fumarate	1	0.29
Fluticasone	1	0.29
Furosemide	1	0.29
Gabapentin	1	0.29
Insulin glargine	1	0.29
Ipratropium	1	0.29
Lamotrigine	1	0.29
Mesalazine	1	0.29
Metronidazole	1	0.29
Montelukast	1	0.29
Nitrofurantoin	1	0.29
Nitrofurantoin XR	1	0.29
Olanzapine	1	0.29
Omeprazole	1	0.29
Oseltamivir	1	0.29
Progesterone	1	0.29
Quetiapine	1	0.29
Sulfasalazine	1	0.29
Tacrolimus	1	0.29
Tinzaparin sodium	1	0.29
Topiramate	1	0.29
Ursodeoxycholic acid	1	0.29
**Total**	**348**	**100.0**

3
